# Estimation for running time and energy losses due to unproductive stops at bus stations in urban-rural traffic corridors

**DOI:** 10.1371/journal.pone.0290903

**Published:** 2023-09-18

**Authors:** Xiuhai Li, Zhan Yu, Peipei Guo, Shaowei Yu

**Affiliations:** 1 Department of Business and Trade, Shandong Vocational College of Light Industry, Zi’bo, Shandong, China; 2 College of Transportation Engineering, Chang’an University, Xi’an, Shanxi, China; Beijing University of Posts and Telecommunications, CHINA

## Abstract

To provide data support for developing fixed-route DRT based on FRT to reduce operating costs inside base routes in urban-rural traffic corridors, this paper estimated running time and energy losses due to unproductive stops at bus stations in urban-rural traffic corridors. Firstly, 14 urban-rural bus routes without ticket sellers in Xi’an are selected to demonstrate the universality of unproductive stops at bus stations. Secondly, a model for estimating running time and energy losses based on the VT-CPFM model is developed. Finally, running time and energy losses due to unproductive stops in two representative urban-rural traffic corridors are estimated. Estimated results show that the average running time loss ratios of different rounds in Routes 332, 333, 335, 338 and G1 range from 8.30% to 17.52% and that average fuel loss ratios range from 9.16% to 13.30%. In addition, the monetary loss in energy consumption of Route G1 in 2019 is estimated to be up to 193213 yuan. This study proves that unproductive stops at bus stations generally exist in urban-rural bus routes and can result in significant running time and energy losses and that developing fixed-route DRT based on FRT leveraging V2I with mobile APP in representative urban-rural traffic corridors is very necessary, which is expected to reduce energy consumption and running time.

## 1. Introduction

Over the recent few decades, China has been experiencing rapid urban-rural integration process and economic development as well as the expansion of college enrollments [1–6]. These processes increase employment opportunities, travel distances and rural-urban commuting frequencies [7–9], which provides opportunities for promoting the implementation of integrated urban and rural bus transit. However, in sparse and low-demand rural areas, at many bus stations except those near universities and companies, passenger demands are meager and dispersive, especially in off-peak hours.

To deal with this problem, many efforts on DRT have been made [10–13]. Pure DRT can provide flexible services desired by passengers, but such a system still tends to be considerably expensive and is mainly limited to specialized operations [14–16]. Hence, experts and scholars shifted the emphasis to FRT [17–21], which can deviate from base routes to serve curb-to-curb requests based on serving regular station-to-station passengers by setting mandatory checkpoints located in high-density demand zones. It is more cost-efficient than pure DRT [22] and more convenient than regular bus routes [23]. However, this service only concentrates on providing flexible services desired by passengers outside base routes as possible through a more cost-efficient network, and it has not yet considered how to reduce operating costs inside base routes.

On the one hand, in urban-rural traffic corridors, except bus stations in urban areas and those near universities and companies, neither boarding nor alighting can be commonly found at the other stations, which can lead to unproductive stops in bus routes adopting local service. However, the operator makes each bus stop at every stop to reduce the number of complaints. Unproductive stops at bus stations can waste running time and energy [24]. In contrast, as far as we know, these losses haven’t been quantitatively estimated, which is very important for policy design and the development and application of new technologies. On the other hand, urban-rural traffic corridors are good candidates for operating base routes of FRT. In addition, emerging V2X communication makes it possible to obtain real-time passenger boarding and alighting information at bus stations. These inspire us to develop fixed-route DRT to reduce operating costs inside base routes traveling through urban-rural traffic corridors, based on FRT leveraging V2I with mobile APP more cost-efficiently and similar to elevator traffic operation. However, the forceful necessity of developing such a system has not been quantitatively provided.

This paper aims at revealing the improper operation management of bus companies, estimating running time and energy losses due to unproductive stops at bus stations of bus routes in representative urban-rural traffic corridors, and providing the forceful necessity of developing fixed-route DRT. The remainder of this paper is organized as follows. The universality of unproductive stops at bus stations in urban-rural bus transit, including data collection and descriptive analysis, is demonstrated in Section 2. Section 3 gives an estimation model for running time and energy losses based on the VT-CPFM model. Running time and energy losses in bus routes of two representative urban-rural traffic corridors are estimated in Section 4. Finally, conclusions are given in Section 5.

## 2. Demonstrating the universality of unproductive stops

Some urban-rural bus routes in Xi’an have been involved in demonstrating the universality of unproductive stops at urban-rural bus stations.

**Fig 1:**
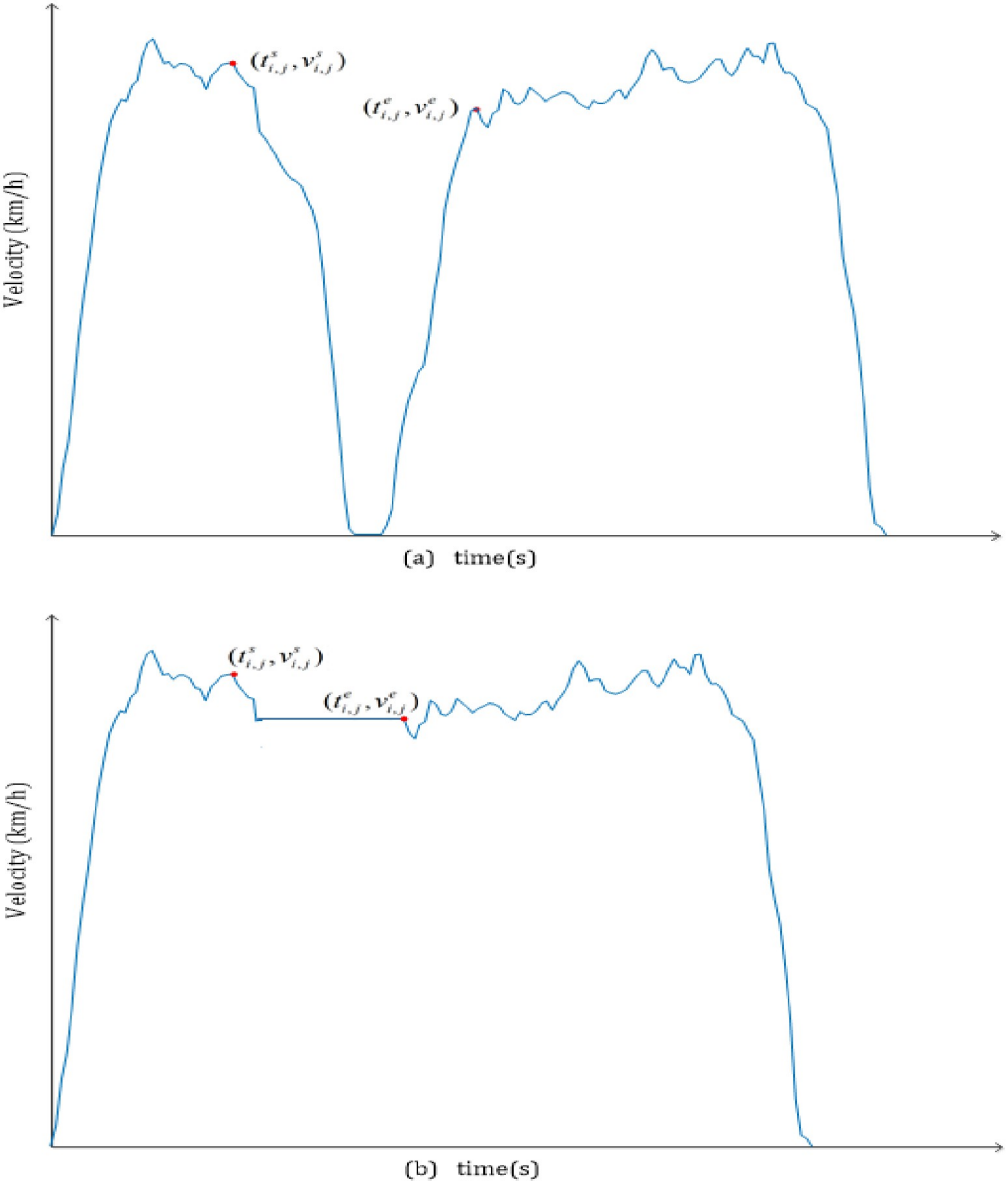
The schematic diagram of Pattern 1 and Pattern 2 (source: Authors).

As the northwest national central city and the capital of Shaanxi Province, Xi’an City has also experienced a rapid urbanization process, economic development, and expansion of college enrollments [25, 26]. It administers 13 county-level divisions, including 11 districts and 2 counties (see Fig 1). According to urbanization degree, they can be divided into three types: the urban area, the suburban area and the exurban area. The urban area includes Xincheng, Beilin, Lianhu and Yanta. The suburban area includes Chang’an, Weiyang and Baqiao. The exurban area includes Huyi, Zhouzhi, Lantian, Lintong, Gaoling and Yanliang.

Fourteen urban-rural bus routes without ticket sellers are selected as study examples. These routes travel through seven districts and one county, including Xincheng, Beilin, Lianhu, Yanta, Chang’an, Weiyang, Lintong, Baqiao and Lantian. Routes bound for Xianyang are not adopted due to the effects of the Xi Xian integration strategy, which have no apparent characteristics of urban-rural bus transit.

### 2.1 Data collection

Data on the spatial distribution of unproductive stops of the selected fourteen bus routes are collected by a survey on the vehicle (on-bus survey), which differs from that was initially used for obtaining the number of boarding and alighting passengers [27, 28]. This survey lasted from July 11 to September 15 in 2019, with one route one weekday, but not all weekdays were fully utilized due to personnel restrictions.

Spatial distribution of unproductive stops as collected by completinga series of survey forms. In these forms, arrival time and the number of boarding and alighting at each station were recorded. Arrival time was measured in minutes. In addition, without or with the ticket seller was concerned. Please note that data of entire bus routes were recorded. For simplicity, the survey form is not listed in this paper. Note that these selected routes have no voluntary skip-stops due to local service.

Data on trajectories were also collected together for further estimating running time and energy losses with the help of GPS-IMU and a Think Pad laptop.

The GPS-IMU device integrates GPS with Inertial Navigation System; the output rate of this device was set as 10HZ. And the original data were processed into second-level trajectories, including the values of speed, acceleration, longitude, latitude, altitude, GPS height, GPS yaw, etc. For simplicity, these data are not listed in this paper.

### 2.2 Descriptive analysis

In this section, the proportion of unproductive stops to all passing stations can be defined as the unproductive stop ratio. Unproductive stop ratios of up and down trips in different rounds of fourteen routes are organized in Table 1.

**Table 1:** Unproductive stop ratios of fourteen bus routes.

No.	Route	Round 1		Round 2		Round 3		Round 4	
		Up	Down	Up	Down	Up	Down	Up	Down
**1**	332	0.400	0.587	0.578	0.413	0.422	0.543	0.422	0.522
**2**	333	0.220	0.160	0.240	0.480	0.320	0.380	0.220	0.240
**3**	334	0.417	0.571	0.583	0.571	0.361	0.543	0.528	0.371
**4**	335	0.500	0.310	0.321	0.345	0.429	0.517	0.214	0.552
**5**	273	0.414	0.207	0.414	0.414	0.379	0.276	0.207	0.172
**6**	901	0.306	0.231	0.286	0.250	0.163	0.250	0.252	0.244
**7**	241	0.579	0.111	0.211	0.056	0.263	0.167	0.263	0.167
**8**	841	0.158	0.158	0.263	0.263	0.368	0.158	0.211	0.211
**9**	G1	0.305	0.241	0.356	0.396	0.356	0.362	0.203	0.241
**10**	240	0.083	0.147	0.389	0.059	0.112	0.118	0.222	0.059
**11**	G2	0.595	0.378	0.432	0.162	0.270	0.378	0.405	0.432
**12**	4–03	0.296	0.555	0.444	0.292	0.185	0.333	0.148	0.555
**13**	338	0.714	0.667	0.571	0.750	0.714	0.458	0.571	0.500
**14**	270	0.175	0.250	0.150	0.025	0.075	0.075	0.110	0.117

Table 1 shows that unproductive stop ratios in up trips range from 0.025 to 0.714 and those in down trips range from 0.075 to 0.750. Nevertheless, obtaining more detailed results without the probability distribution of unproductive stop ratios is difficult.

Further more, unproductive stop ratios can be grouped by eight intervals and counted; frequency in different intervals and their average values of up and down trips in different rounds are organized in Table 2.

**Table 2:** Frequency in different intervals.

Interval	Round 1		Round 2		Round 3		Round 4		Mean	
	Up	Down	Up	Down	Up	Down	Up	Down	Up	Down
**0.0–0.1**	7.14%	0.00%	0.00%	21.43%	7.14%	7.14%	0.00%	7.14%	3.57%	8.93%
**0.1–0.2**	14.29%	28.57%	7.14%	7.14%	21.43%	21.43%	14.29%	21.43%	14.29%	19.64%
**0.2–0.3**	14.29%	28.57%	28.57%	21.43%	14.29%	14.29%	57.14%	28.57%	28.57%	23.21%
**0.3–0.4**	21.43%	14.29%	21.43%	14.29%	35.71%	28.57%	0.00%	7.14%	19.64%	16.07%
**0.4–0.5**	21.43%	0.00%	21.43%	21.43%	14.29%	7.14%	14.29%	14.29%	17.85%	10.71%
**0.5–0.6**	14.29%	21.43%	21.43%	7.14%	0.00%	21.43%	14.29%	21.43%	12.50%	17.85%
**0.6–0.7**	0.00%	7.14%	0.00%	0.00%	0.00%	0.00%	0.00%	0.00%	0.00%	1.79%
**0.7–0.8**	7.14%	0.00%	0.00%	7.14%	7.14%	0.00%	0.00%	0.00%	3.57%	1.79%

Table 2 shows that most unproductive stop ratios are distributed in the interval from 0.2 to 0.6, and a few are distributed in the other intervals. It can also be found that the cumulative frequency of unproductive stop ratios distributed in the interval from 0.2 to 0.6 is respectively 71.4%, 64.29%, 92.86%, 64.29%, 64.29%, 71.4%, 85.71% and 71.4% and that their average values also obey this law. This means there are high-proportioned unproductive stops in these fourteen bus routes.

Therefore, high-proportioned unproductive stops generally exist in these selected urban-rural bus routes. There may be massive potential in reducing running time and energy consumption by adopting new technologies or operational strategies. However, the effects of high-proportioned unproductive stops on running time and energy consumption have not been quantitatively estimated.

## 3. Estimation model

To estimate running time and energy losses due to unproductive stops at bus stations, models for estimating running time loss and for estimating energy loss are proposed. In this section, two kinds of service patterns are concerned. One is the local service pattern, and the other is the limited service pattern with exact and real-time skip-stops. For convenience, the former is called Pattern 1 and the latter is called Pattern 2.

In Pattern 1, buses need to adjust the speed from one average running speed to zero, stop for a moment for boarding and alighting, and then speed up to another average running speed when approaching every intermediate bus station (see Fig 1(a)). These behaviors can waste running time and energy at those bus stations without boarding or alighting. In Pattern 2, buses pass through those stations without boarding or alighting at a constant velocity. The constant velocity is supposed to be the smaller instantaneous velocity between the starting time of deceleration and that at the ending time of acceleration (see Fig 1(b)).

In Fig 1, $t_{i,j}^{s}$ is the starting time of deceleration of the Bus *i* at the Station *j*, and $t_{i,j}^{e}$ is the ending time of acceleration in Pattern 1. $t_{i,j}^{es}$ is the time of the Bus *i* at Station *j* at the location of $D_{i,j}^{e}$ in Pattern 2. $v_{i,j}^{e}$ is the instantaneous velocity of Bus *i* at Station *j* at the time of $t_{i,j}^{e}$, and $v_{i,j}^{s}$ is the instantaneous velocity at the time of $t_{i,j}^{s}$.

### 3.1 Model for estimating running time loss

Running time losses due to unproductive stops at bus stations in a one-way trip can be defined as the difference between the running time in Pattern 1 and Pattern 2.

Firstly, the running time of the Bus *i* at the Station *j* in Pattern 1 is defined as

$$T_{i,j}^{1}=t_{i,j}^{e}-t_{i,j}^{s}$$ (1)

Secondly, the running time of the Bus *i* at the Station *j* in Pattern 2 is defined as

$$T_{i,j}^{\text{2}}=t_{i,j}^{es}-t_{i,j}^{s}$$ (2)

$t_{i,j}^{es}$ can be determined by

$$t_{i,j}^{es}=\left(D_{i,j}^{e}-D_{i,j}^{s}\right)/\left[\text{min}\left(v_{i,j}^{s},v_{i,j}^{e}\right)\right]\text{+}t_{i,j}^{s}$$ (3)

Thirdly, the running time loss of the Bus *i* at the Station *j* due to an unproductive stop is defined as

$$T_{i,j}^{loss}=T_{i,j}^{\text{1}}-T_{i,j}^{\text{2}}$$ (4)

Fourthly, the running time loss of a Bus *i* on a one-way trip is defined as

$$T_{i}^{loss}{}^{}=\sum_{j=1}^{L}T_{i,j}^{loss}$$ (5)

Finally, the running time loss ratio of a Bus *i* on a one-way trip is defined as

$$PT_{i}^{loss}=T_{i}^{loss}/\sum_{j=1}^{L}T_{i,j}^{2}$$ (6)

Where $D_{i,j}^{e}$ is the location of the Bus *i* at Station *j* at the time of $t_{i,j}^{e}$, and $D_{i,j}^{s}$ is the location at the time of $t_{i,j}^{s}$. $T_{i,j}^{\text{1}}$ is the running time of the Bus *i* at the Station *j* in Pattern 1, and $T_{i,j}^{\text{2}}$ is that in Pattern 2. $T_{i,j}^{loss}$ is the running time loss of the Bus *i* at the Station *j* due to unproductive stop. $T_{i}^{loss}$ is the running time loss of Bus *i* in a one-way trip. $PT_{i}^{loss}$ is the running time loss ratio of Bus *i* in one-way trip. *L* is the total number of bus stations in one bus route.

### 3.2 Model for estimating energy loss

Energy losses due to unproductive stops at bus stations in a one-way trip can be defined as the difference between the energy consumption in Pattern 1 and Pattern 2.

Firstly, the energy consumption of the Bus *i* at the Station *j* in Pattern 1 is defined as

$$F_{i,j}^{1}=\int_{t_{i,j}^{s}}^{t_{i,j}^{e}}FC_{i}\left(t\right)dt$$ (7)

Secondly, the energy consumption of the Bus *i* at the Station *j* in Pattern 2 is defined as

$$F_{i,j}^{\text{2}}=\int_{t_{i,j}^{s}}^{t_{i,j}^{es}}FC_{i}\left(t\right)dt$$ (8)

Thirdly, the energy loss of the Bus *i* at the Station *j* due to unproductive stops is determined by

$$F_{i,j}^{loss}=F_{i,j}^{\text{1}}-F_{i,j}^{\text{2}}$$ (9)

Fourthly, the energy loss of a Bus *i* on a one-way trip is defined as

$$F_{i}^{loss}=\sum_{j=1}^{L}F_{i,j}^{loss}$$ (10)

Finally, the energy loss ratio of a Bus *i* on a one-way trip is defined as

$$PF_{i}^{loss}=\sum_{j=1}^{L}F_{i,j}^{loss}/\sum_{j=1}^{L}F_{i,j}^{2}$$ (11)

Where $F_{i,j}^{\text{1}}$ is the energy consumption of the Bus *i* at the Station *j* in Pattern 1, and $F_{i,j}^{\text{2}}$ is that in Pattern 2. *FC*_*i*_(*t*) is the energy consumption rate of the Bus *i*. $F_{i,j}^{loss}$ is the energy loss of the Bus *i* at the Station *j* with unproductive stop. $F_{{}^{i}}^{loss}$ and $PF_{{}^{i}}^{loss}$ are respectively the energy loss and the energy loss ratio of a Bus *i* in a one-way trip. *N* is the total number of one-way trips.

On the one hand, vehicles of the selected fourteen bus routes use different energy like petrol, natural gas and electricity, and it isn’t easy to find a universal energy consumption model. On the other hand, there was a significant variation of the dynamic characteristic among different buses, and kinetic parameters are not universal. Therefore, an approximate method is given to estimate energy losses.

Firstly, the VT-CPFM model for conventional diesel buses [29] is adopted as the alternative, based on the basic assumption that fuel consumption is proportional to energy consumed by a vehicle, which is the only fuel consumption model for diesel buses found in the current literature. Formulations of the VT-CPFM model are given as

$$FC_{i}\left(t\right)=\left\{\begin{array}{l} \beta_{0}+\beta_{1}P_{i}\left(t\right)+\beta_{2}{\left[P_{i}\left(t\right)\right]}^{2}\;\forall P_{i}\left(t\right)\geq 0\\ \beta_{0}\;\forall P_{i}\left(t\right)<0 \end{array}\right.$$ (12)

$$P_{i}\left(t\right)=\left(\frac{R_{i}\left(t\right)+\left(1+\lambda +0.0025\xi {\left(v_{i}\left(t\right)\right)}^{2}\right)m_{i}a_{i}\left(t\right)}{3600\eta_{d}}\right).v_{i}\left(t\right)$$ (13)

$$R_{i}\left(t\right)=\frac{\rho }{25.92}C_{d}C_{h}A_{f}{\left[v_{i}\left(t\right)\right]}^{2}+m_{i}g\frac{C_{r}}{1000}\left(c_{1}v_{i}\left(t\right)+c_{2}\right)+m_{i}gG\left(t\right)$$ (14)

$$C_{h}=1-0.085H$$ (15)

Where *P*_*i*_(*t*) is the instantaneous power. *R*_*i*_(*t*) is the resistance force. *m*_*i*_ is the bus mass. *a*_*i*_(*t*) and *v*_*i*_(*t*) are, respectively, the instantaneous acceleration and velocity. *β*_n_, *β*_1_ and *β*_2_ are the bus-specific model coefficients. *ξ* The term related to gear ratio is assumed to be zero due to the lack of gear data. *η*_*d*_ is the driveline efficiency. *ρ* is the air density at sea level. *C*_*d*_ is the vehicle drag coefficient. *C*_*h*_ is the altitude correction factor. *H* is the altitude in the unit of km. *A*_*f*_ is the frontal area of buses. *C*_*r*_, *c*_1_ and *c*_2_ are rolling resistance parameters. *G*(*t*) is the road grade.

Secondly, all buses are assumed to be the same, and the dynamic parameters of Bus 6323 [29] are used as the alternative to calculating fuel consumption. The road grade is supposed as 0, and the Internet obtains the altitude of Xi’an city. The other parameters in the fuel consumption model are listed in Table 3.

**Table 3:** Parameters in the fuel consumption model.

Parameter	Value	Parameter	Value	Parameter	Value	Parameter	Value
*β*_0_	1.230e-03	*ξ*	0	*ρ*	1.2256 kg/m^3^	*A*_*f*_	6.824 m^2^
*β*_1_	1.125e-04	*m*_*p*,*n*_	17996 kg	*C*_*d*_	0.8	*C*_*r*_	1.25
*β*_2_	4.154e-07	*g*	9.8066 m/s^2^	*C*_*h*_	0.9932	*c*_1_	0.0328
*λ*	0.1	*η*_*d*_	0.95	*H*	0.556 km	*c*_2_	4.575

Finally, fuel losses and loss ratios due to unproductive stops at bus stations are first estimated. Then monetary losses due to unproductive stops are estimated using fuel loss ratios and data on energy costs acquired from bus transit operators. Note that this method for energy consumption estimation can be applied to electric or other vehicle types.

## 4. Results and analysis

Tall buildings, high trees and overpasses in urban areas may result in low positioning accuracy and data loss, which are unsuitable for estimating energy losses based on second-level velocity and acceleration. In addition, traffic corridors may be good candidates to promote connected and automated bus transit systems based on V2X technology for high-efficiency Integrated Corridor Management [30]. Therefore, the rural areas of urban-rural traffic corridors deserve more attention.

### 4.1 Running time loss estimation

GPS-IMU measures the running time of different one-way trips in Pattern 1, and that in Pattern 2 is calculated by Eqs (2) and (3). Running times of different one-way trips of Routes 332, 333, 335, 338 and G1 in Corridor 1 and Corridor 2 are organized $T_{i}^{1}/T_{i}^{2}$ from Row 1 to Row 6 in Table 4. The average value of different rounds of each bus route in Corridor 1 and Corridor 2 is also listed in the last row. $T_{i}^{1}$ means the running time of the Bus *i* in Pattern 1 and that of Bus *i* in Pattern 2. They are measured in seconds and rounded to the nearest whole number.

**Table 4:** Running time of different trips in Pattern 1 and Pattern 2.

Round	Trip	332	333	335	338	G1
**Round 1**	**Up**	1621/1473	1528/1344	1569/1463	1585/1403	1381/1143
	**Down**	1716/1596	1548/1419	1640/1442	1554/1198	1736/1526
**Round 2**	**Up**	1582/1385	1660/1541	1571/1505	1663/1450	1562/1304
	**Down**	1603/1403	1603/1456	1569/1414	1932/1740	1574/1380
**Round 3**	**Up**	1678/1481	1570/1447	1797/1670	1737/1499	1592/1433
	**Down**	1712/1512	2109/1980	1622/1532	1569/1299	1906/1728
**Mean**	**Round**	1652/1475	1670/1531	1628/1504	1673/1432	1625/1419

Table 4 shows that the running time of different one-way trips of these selected bus routes in Pattern 1 is obviously more remarkable than those in Pattern 2, and so are the average values of different rounds of different bus routes. It can also be seen that running time varies in different one-way trips of each bus route in Pattern 1 and Pattern 2, which results from different traffic states, traffic signals, and driving behaviors.

However, it is challenging to explicitly find the difference between Pattern 1 and Pattern 2 in Table 4. To this end, running time losses and loss ratios of different one-way trips of Routes 332, 333, 335, 338 and G1 are estimated by using the model (1)-(6), and they are organized in the form of $T_{i}^{loss}/PT_{i}^{loss}$ from Row 1 to Row 6 in Table 5. The average value of different rounds of each bus route is also listed in the last row. $T_{i}^{loss}$ means the running time loss of a Bus *i* on a one-way trip, measured in seconds and rounded to the nearest whole number. $PT_{i}^{loss}$ means the loss ratio is measured to be accurate in 2 decimal places.

**Table 5:** Running time loss and loss ratios of different trips.

Round	Trip	332	333	335	338	G1
**Round 1**	**Up**	148/10.05%	184/13.69%	106/7.25%	182/12.97%	238/20.82%
	**Down**	120/7.52%	129/9.09%	198/13.73%	356/29.72%	210/13.76%
**Round 2**	**Up**	197/14.22%	119/7.72%	66/4.39%	213/14.69%	258/19.79%
	**Down**	200/14.26%	147/10.10%	155/10.96%	192/11.04%	194/14.06%
**Round 3**	**Up**	197/13.30%	123/8.50%	127/7.61%	238/15.88%	159/11.10%
	**Down**	200/13.23%	129/6.52%	90/5.88%	270/20.79%	178/10.30%
**Mean**	**Round**	177/12.10%	139/9.27%	124/8.30%	242/17.52%	206/14.97%

Table 5 shows that running time losses vary in different one-way trips of each bus route due to different unproductive stop ratios and that loss ratios also vary in different one-way trips due to differences in the running time of Pattern 2 and running time losses. It can also be seen that $PT_{i}^{loss}$ ranges from 4.39% to 29.72%, and that average running time loss ratios of different rounds of Routes 332, 333, 335, 338 and G1 are respectively 12.10%, 9.27%, 8.30%, 17.52% and 14.97%.

### 4.2 Fuel loss estimation

Fuel consumptions in Pattern 1 and Pattern 2 are calculated by using the measured second-level velocity and acceleration based on the model (12)-(15). And then, fuel losses and loss ratios of different one-way trips of Routes 332, 333, 335, 338 and G1 are obtained using the model (7)-(11), and they are organized in the form of $F_{i}^{loss}/PF_{i}^{loss}$ from Row 1 to Row 6 in Table 6. The average value of different rounds of each bus route is also listed in the last row. $F_{i}^{loss}$ means the fuel loss of a Bus *i* in a one-way trip, measured in milliliters. $PF_{i}^{loss}$ means the loss ratio, which is accurate in 2 decimal places. Please note that the trajectory data of Route 338 were damaged, and the corresponding results can’t be given in the following section.

**Table 6:** Fuel loss and loss ratios of different trips.

Round	Trip	332	333	335	338	G1
**Round 1**	**Up**	439/9.40%	496/12.12%	469/10.43%	---/---	438/11.64%
	**Down**	352/7.35%	508/10.90%	465/11.40%	---/---	849/17.26%
**Round 2**	**Up**	586/10.97%	385/9.28%	128/2.91%	---/---	474/12.61%
	**Down**	586/12.25%	373/8.94%	549/13.45%	---/---	784/15.93%
**Round 3**	**Up**	586/12.53%	444/8.93%	481/10.79%	---/---	292/7.76%
	**Down**	586/12.25%	477/8.99%	275/5.96%	---/---	718/14.61%
**Mean**	**Round**	523/10.79%	447/9.86%	395/9.16%	---/---	593/13.30%

Table 6 shows that fuel losses vary in different one-way trips of each bus route due to different unproductive stop ratios and loss ratios also vary in different one-way trips due to differences in fuel consumption of Pattern 2 and fuel losses. It can also be seen that ranges from 2.91% to 17.26%, and that the average fuel loss ratios of different rounds of these routes are 10.79%, 9.86%, 9.16% and 13.30%, respectively. This means that high-proportioned unproductive stops at bus stations can result in significant fuel losses.

Based on the above estimation, it is challenging to estimate explicitly monetary losses in fuel consumption due to unproductive stops at bus stations. Thus, monetary losses in fuel consumption are further estimated using bus transit operators’ average fuel loss ratios and monthly energy costs of Routes 332, 333, 335, 338 and G1 in 2019. It is assumed that each route’s fuel loss ratio did not vary throughout the year because of the absence of daily data on unproductive stop ratios. Energy costs and monetary losses are organized in the form of *EC*/*EL* Table 7. *EC* and *EL*, respectively, mean energy costs and monetary losses rounded up to the nearest whole number and in the unit of yuan.

**Table 7:** Estimated monetary losses in 2019.

Month	332	333	335	338	G1
**January**	106857/10407	125908/11300	23729/1990	---/---	132750/15583
**February**	89513/8718	99394/8921	19824/1663	---/---	121281/14237
**March**	108673/10584	102754/9222	54791/4595	---/---	137542/16146
**April**	113619/11066	95279/8551	88241/7401	---/---	128453/15079
**May**	121387/11822	101308/9093	95264/7990	---/---	134138/15746
**June**	123041/11983	107544/9652	98955/8300	---/---	119959/14082
**July**	131392/12797	119002/10681	109332/9170	---/---	120339/14126
**August**	130260/12686	114732/10297	106070/8896	---/---	117224/13761
**September**	124298/12106	100404/9011	96298/8077	32986/---	119457/14023
**October**	115498/11249	102483/9198	94817/7953	46965/---	143050/16792
**November**	128996/12563	112063/10058	104707/8782	48419/---	174112/20439
**December**	126220/12293	130217/11687	116553/9776	53436/---	197636/23200
**Year**	1419754/138272	1311088/117671	1008581/84591	181806/---	1645941/193213

In Table 7, it can be seen that the monetary losses of Routes 332, 333, 335, 338 and G1 in February are the lowest because of winter vacation, and that of Route 335 is much less than those of the other bus routes because winter vacation of two colleges at departure and terminal stations began relatively earlier. It can also be found that monthly monetary losses range from 1663 yuan to 23200 yuan and that yearly monetary losses of these routes are respectively 138272 yuan, 117671 yuan, 84591 yuan and 193213 yuan. This means that high-proportioned unproductive stops at bus stations can result in significant monetary losses in fuel consumption.

The above estimations for running time and energy losses indicate that unproductive stops at bus stations can result in significant running time, energy losses, and monetary losses in fuel consumption. These prove the improper operation in the current situation and provide forceful data support to developing fixed-route DRT based on FRT and eco-driving [30–32] in urban-rural traffic corridors is essential, which is expected to reduce energy consumption and running time.

## 5. Conclusions

Compared with route planning of FRT, this paper reveals that unproductive stops generally exist in these selected urban-rural bus routes, and that unproductive stops at bus stations can result in significant running time and energy losses as well as monetary losses in fuel consumption.

Futher more, this paper proves that developing fixed-route DRT based on FRT leveraging V2I with mobile APP in urban-rural traffic corridors is very necessary for reducing unproductive stop ratios.

This topic is very interesting., however, two limitations still exist in this paper in terms of current technology: (1) whether unproductive stop ratios vary at different periods in a day and different directions or not can’t be obtained in the absence of data in an entire day due to personnel constraints; (2) Not all urban-rural bus routes are quantitatively estimated due to cost constraints. Big data and Internet of Vehicles Technology will investigate these issues, which are expected to be applied to connected and automated buses and transit network design in the near-future study. These issues will be investigated in the near future.

## Supporting information

S1 Data (RAR)
